# Circular STAG2 RNA Modulates Bladder Cancer Progression via miR-145-5p/TAGLN2 and Is Considered as a Biomarker for Recurrence

**DOI:** 10.3390/cancers16050978

**Published:** 2024-02-28

**Authors:** Chris Du, Wayne C. Waltzer, Jeremy E. Wilusz, Massimiliano Spaliviero, Frank Darras, Victor Romanov

**Affiliations:** 1 Department of Urology, Renaissance School of Medicine, SUNY at Stony Brook, Stony Brook, NY 11794, USA; chris.du@stonybrookmedicine.edu (C.D.); wayne.waltzer@stonybrookmedicine.edu (W.C.W.); massimiliano.spaliviero@stonybrookmedicine.edu (M.S.); frank.darras@stonybrookmedicine.edu (F.D.); 2 Department of Biochemistry & Molecular Pharmacology, Therapeutic Innovation Center, Baylor College of Medicine, Houston, TX 77030, USA; jeremy.wilusz@bcm.edu

**Keywords:** circular RNA, liquid biopsy, bladder cancer, STAG2, miR-145-5p, TAGLN2

## Abstract

**Simple Summary:**

Bladder cancer (BCa) is a major urologic malignancy with a high potential for death-threatening complications. Successful treatment relies on timely diagnostics and prediction of clinical outcomes. Molecular markers can be helpful for earlier diagnosis and for making treatment decisions. The biomarker landscape for BCa is not satisfactory yet. The discovery of novel diagnostic indicators for making treatment decisions is critical. Biological fluids are the most convenient and reliable source of these molecules. Circular RNAs (CircRNAs), special non-coding RNAs with a stable structure that are formed by a unique backsplicing process, recently became popular candidates for biomarkers in cancer biology. CircRNAs are widely distributed within the body including biological fluids. Their biological functions include gene regulation via sponging microRNAs, another type of small regulatory RNA. Our study characterized the circRNA hsa_circ_0139697 (circSTAG(16–25)) derived from exons 16 to 25 of STAG2, a gene known to play an important role in BCa biology that can serve as a biomarker predicting recurrence. We found that circSTAG2(16–25) could promote the proliferation, migration, and invasion of BCa cells by binding miR-145-5p, which causes the upregulation of the potential onco-gene TAGLN2. In addition, circSTAG(16–25) can serve as a biomarker for recurrence prediction.

**Abstract:**

The current study aimed to elucidate the regulatory mechanisms of the circRNA hsa_circ_0139697 (circSTAG2(16–25)) in BCa and to consider the opportunity of using circSTAG2(16–25) isolated from BCa patient urine as a marker for disease development prediction. The selection of this circRNA was determined by the special role of its parental gene STAG2 in BCa biology. The circRNA hsa_circ_0139697 was chosen from 25 STAG2 circRNAs due to its differential expression in the urine of BCa patients and healthy volunteers. Higher levels of circSTAG2(16–25) were detected in urine samples obtained from patients with recurrent tumors. A higher expression of circSTAG2(16–25) was also detected in more tumorigenic BCa cell lines. The overexpression of circSTAG2(16–25) in BCa cells induced the elevation of proliferation, motility, and invasion. To study the mechanisms of circSTAG2(16–25) activity, we confirmed that circSTAG2(16–25) can bind miR-145-5p in vitro as was predicted by bioinformatic search. miR-145-5p was shown to suppress some genes that promoted BCa progression. One of these genes, TAGLN2, encodes the protein Transgelin 2, which plays a role in BCa cell motility and invasion. Therefore, the possible mechanism of action of circSTAG2(16–25) could be sponging the tumor suppressor miR-145-5p, which results in activation of TAGLN2. In addition, circSTAG2(16–25) might be considered as a potential biomarker for recurrence prediction.

## 1. Introduction

Bladder cancer is the second most common malignancy of the urinary system, and it is a heterogeneous disease with significant diagnostic, therapeutic, and prognostic problems. Early detection signifies a better disease prognosis; thus, minimally invasive diagnostic options are needed to improve patient outcomes [1]. Molecular markers can provide the opportunity to diagnose a bladder tumor early, identify patients who are at risk of recurrence, and/or predict how tumors will respond to therapeutic approaches [2]. A patient’s urine can be considered a reliable and convenient source of such biomarkers. DNA, RNA and protein molecules derived from the urine of BCa patients have previously been considered as diagnostic and prognostic markers for BCa [3].

Circular RNAs (circRNAs), which have covalently linked ends, have attracted increasing attention because of their potential for biological activity in different clinical fields, including cancer biology [4,5]. It was shown that circRNAs isolated from urine can serve as biomarkers for different urological diseases [6]. CircRNAs are generated during pre-mRNA splicing when a downstream 5′ splice site is joined to an upstream 3′ splice site [7,8,9]. Most mature circRNAs are expressed at low levels, but some have known physiological functions including in gene regulation, e.g., by binding to microRNAs, and/or accumulating to higher levels than their associated linear mRNAs [10]. This is because circRNAs are naturally resistant to riboexonucleases and more stable than linear RNA molecules, making them potentially very promising biomarkers. One possible mechanism of circRNA action is sponging or sequestering another type of active small RNA, microRNA. MicroRNAs suppress the activity of mRNAs that regulate the progression of multiple cancers [4,11].

For this study, we focused on a circRNA synthesized from the human STAG2 gene that played a perceptible role in BCa biology. Stromal antigen 2 (STAG2) protein encoded by the STAG2 gene is a subunit of the cohesin complex, which regulates sister chromatid separation during cell division. STAG2 is one of the most mutated genes in BCa. Most mutations (non-sense, frameshift, and splicing) are inactivating, and induce complete loss of protein expression in non-muscle invasive BCa (NMIBC). Published data suggest that the loss of STAG2 production is a good prognostic factor in NMIBC [12,13,14,15,16,17,18].

The current study characterized in detail one of the circRNAs derived from STAG2. CircSTAG2(16–25) was differentially detected in urine derived from BCa patients and healthy volunteers. It further was found at higher levels in urine derived from patients with recurrent tumors as well as more abundant in higher tumorigenic BCa cell lines. Our results suggest that circSTAG2(16–25) may regulate the behavior of cancer cells by sponging miR-145-5p, a microRNA that is known to possess tumor suppressor functions in BCa [19,20,21,22,23,24,25]. One of the downstream miR-145-5p targets is TAGLN2, which is known to promote motility and invasion of BCa cells [20]. Therefore, we propose that circSTAG2(16–25) can regulate cancer cell behavior via miR-145-5p and TAGLN2.

## 2. Materials and Methods

### 2.1. Cell Culture

Human BCa cells (T24 and 5637) and human uroepithelial immortalized UROtsa cells were obtained from ATCC (Manassas, VA, USA) T24 and 5637 cells were cultivated in RPMI 1640 medium (Gibco, Grand Island, NY, USA) containing 10% fetal bovine serum (FBS), and UROtsa cells were cultivated in DMEM medium (Gibco, USA) containing 10% FBS. All cells were cultured at 37 °C in a 5% CO_2_ incubator.

### 2.2. Patient Specimens

Eighty urine samples and seven matched tumors were collected from patients diagnosed with bladder cancer from March 2022 to September 2023. Twenty additional urine specimens were obtained from healthy donors from March 2022 to August 2023. Fifty-three examined patients were male. The age range was 53–95 years. The pathological types were confirmed by professional pathologists. Written informed consent was acquired from all patients, and this study was approved by the IRB committee of Stony Brook School of Medicine.

### 2.3. RNA Extraction and RT-qPCR

Total RNA of tissues and cells was extracted with TRIzol Reagent (Invitrogen, Waltham, MA, USA) following the manufacturer’s instructions. RNA from urine was extracted with Urine Cell-free circulating RNA kit (Norgen Biotech, Thorold, ON, Canada) or RNAzol reagent (MRC, Houston, TX, USA). After that, RNA was reverse transcribed into cDNA with Evo cDNA kit (BioVision, Milpitas, CA, USA) using random primers or miR-145-5p stem-loop primer: GTCGTATCCAGTAGGGTCCGAGGTATTCGCACTGGATAGACAGGGATTCCTG. All primers were synthesized by IDT (Coralville, IA, USA) and sequences of primers for RT-qPCR are provided in Table 1. The circular nature of circSTAG2(16–25) was confirmed by Sanger sequencing across the back splicing junction site. All assays were independently repeated at least three times. RT-qPCR was conducted using SYBR Green Master Mix (Biotum, Fremont, CA, USA) on ABI 4300 system (Applied Biosystems, Foster City, CA, USA). U6 and ACTB served as internal references for RT-qPCR.

### 2.4. RNase R Treatment

Total RNA was incubated for 30 min at 37 °C with or without 3 U/μg RNase R (Epicentre Technologies, Mira-Bhayandar, India) followed by RNase R inactivation at 65 °C for 20 min. K^+^ was replaced with Li^+^ in the reaction buffer to improve the efficiency of linear RNA degradation [26]. RNA was then purified using miRNeasy Mini Kit (QIAGEN, Germantown, MD, USA) and eluted in 15 μL of water.

### 2.5. Molecular Cloning and Cell Transfection

CircSTAG2(16–25) siRNA1 (rGrArUrGrArUrGrCrArUrArUrCrArGrGrUrArUrUrGrUrCAA) and siRNA2 (rUrUrGrArCrArArUrArCrCrUrGrArUrArUrGrCrArUrCrArUrCrUrU) were synthesized by IDT based on the sequence of mature circSTAG2(16–25). Both siRNAs encompassed the back splicing junction area. Scrambled negative control siRNA (IDT, USA) served as non-related siRNA (siRNA nr). MiR-145-5p mimics were ordered from Qiagen (USA)-gene globe ID MSY0000851S0 219600. MiR-145-5p Inhibitor was synthesized by IDT (USA) mA/ZEN/mGmGmGmAmUmUmCmC mUmGmGmGmAmAmAmAmCmUmGmGmA/3Z).

The overexpression plasmid pcDNA3.1 (+) ZKSCAN1 Exon MCS vector [27] (Addgene 69901) bearing circSTAG2(16–25) was constructed with NEBuilder HIFI DNA assembly master kit and related software (NEBuilder Assembly Tool, version 2.10.1) (NEB, Ipswich, MA, USA). CircSTAG2(16–25) was inserted between positions 1387 and 1451. Primers used for the cloning: Vector Forward -TTTTTTTTTATACTTCAGGATTACAT TGAGCATGCAGCATACCTTGTGGATAGC; Vector Reverse- CCTTGCTTCTTACCTCCGCGCTGCACTATTATTATCATCATCCTGTTCAATGAAG.

For circSTAG2(16–25): Forward: CGCGGAGGTAAGAAGCAAG; Reverse: TCCTGAAGTATAAAAAAAAAGTCATTAG.

A total of 2 × 10^5^ 5637 or T24 cells were seeded into six-well plates. The next day, small RNAs were transfected into BCa cells with Lipofectamine RNAiMAX reagent (Invitrogen, USA). The circSTAG2(16–25) overexpressing construct and the corresponding control (original ZKSCAN1 MCS vector) were transfected into BCa cells with Viafect (Promega, Fitchburg, WI, USA). After 24 h, RT-qPCR was performed to evaluate transfection efficiency.

### 2.6. Wound-Healing Assay

Wound-healing assays were performed as previously described [28]. Transfected and control cells were cultured to 85–95% confluency in 24-well plates. The scratch was made using a 200 µL tip. Using a microscope camera, photos of the cell scratches were taken at 0 and 24 h. Measurements of the wounded area were performed with Image J software (version 1.54h).

### 2.7. Transwell Invasion Assay

For the Transwell invasion assay, 200 μL serum-free culture medium containing 2 × 10^4^ cells was plated into the upper chamber of a 24-well plate, which was precoated with Matrigel (BD Biosciences, San Jose, CA, USA). 600 μL medium containing 10% FBS was then added to the lower chamber. After incubation for 24 h, the cells that migrated to the membrane of the upper chamber were fixed with 4% paraformaldehyde and stained with 1% crystal violet. The invasive cells were treated with 10% acetic acid and spectrometric absorbance at 525 nm was determined with plate reader [29].

### 2.8. Dual-Luciferase Reporter Assay

The wild-type fragment of circSTAG2(16–25) (circSTAG2 wt), capable of binding to miR-145-5p as predicted with Circinteractome software https://circinteractome.nia.nih.gov/, accessed on 3 April 2023 (Site updated: 2020-01-30, 16:52 EST), and the TAGLN2 sequence that can potentially bind miR-145-5p, as determined by miRDB software (https://mirdb.org/, accessed on 2 June 2023) were inserted into the pmiRGLO plasmid [30] (Addgene 78131). The fragment for binding miR-145-5p was identical for circSTAG2(16–25) and for TAGLN2 (AACTGGA). TGGACTT sequence used as insert for MUT control. To construct dual-luciferase reporter plasmids, we used single-nucleotide oligo bridge technology and NEBuilder HiFi system (NEBuilder Assembly Tool, version 2.10.1) (NEB, Ipswich, MA, USA). Fragments were cloned into the pmiRGLO vector using NheI and XbaI restriction sites. Plasmids were then transfected into BCa cells with or without miR-145-5p mimic using Viafect transfection reagent (Promega, Madison, WI, USA). Twenty-four hours later, firefly luciferase and Renilla luciferase were detected by using a Dual-Lumi™ Luciferase Assay Kit (Promega, USA), and the assays were independently repeated three times.

### 2.9. Statistical Analysis

Experimental data are shown as mean ± standard deviation (SD). Student’s *t*-test was performed to compare differences between unpaired groups. Experimental data were analyzed by GraphPad Prism 5.0.1 software. *p*-value < 0.05 was considered statistically significant.

## 3. Results

### 3.1. CircSTAG2(16–25) Is Up-Regulated in BCa and in More Aggressive Urothelial Carcinoma Cell Lines

**Figure 1:**
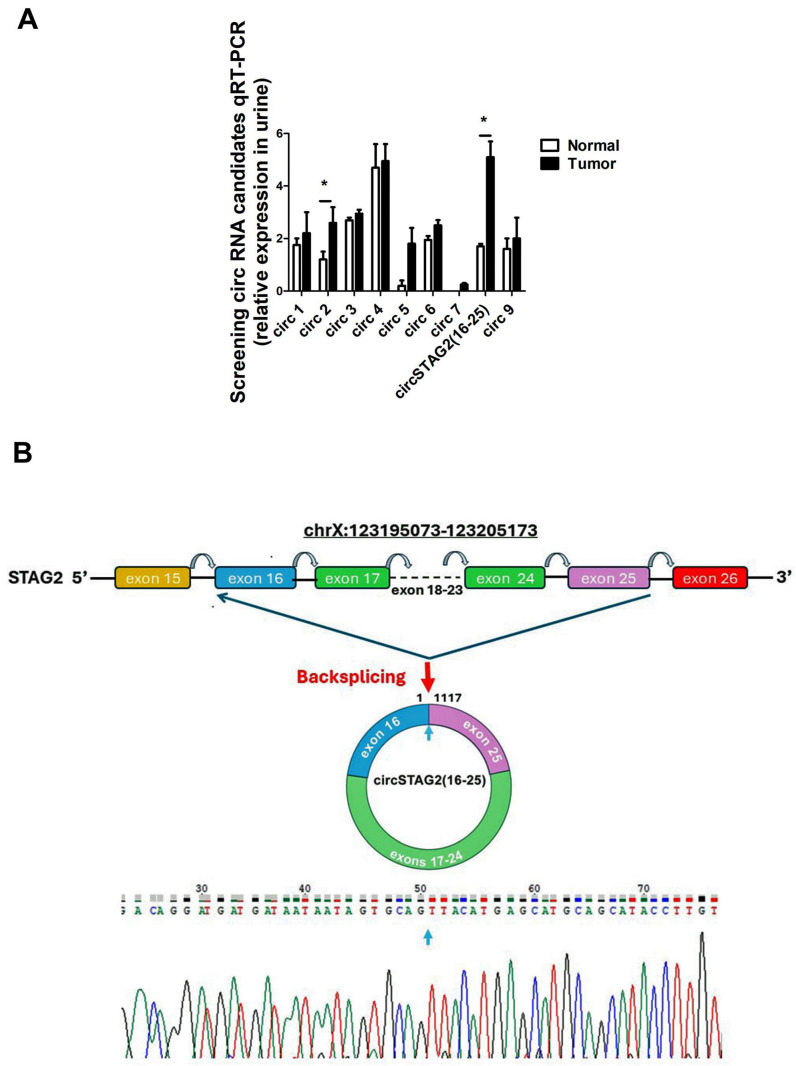
Identification and characterization of circSTAG(16–25) (hsa_circ_0139697) selected for this study. (**A**) Expression of nine selected circRNAs in the urine of three healthy volunteers and three BCa patients (age-matched, two males, one female). * = *p* < 0.05, (**B**) Genomic position of circSTAG2(16–25), which is generated by back splicing of exons 16–25.

The STAG2 gene is highly mutated in BCa (23%). In most NMIBC cases, mutations cause STAG2 to be truncated and these genomic alterations are associated with low recurrence rates [13,16,18,31]. There are no publicly available expression data on circular RNAs derived from the STAG2 gene in BCa or the human urothelium. We thus selected STAG2 for circRNA synthesis and evaluation because of its highly mutated status and specific role in BCa. Twenty-six distinct circRNAs derived from the STAG2 gene have been annotated in circBase [32]. To determine if some of these circRNAs could be detected in urine, we used RT-qPCR and divergent primers that amplify across the back splicing junction to test the expression of 9 of these circRNA candidates. These candidates were selected based on their mature transcript size and their potential ability to bind miRNAs that showed activity in BCa. All tested circSTAG2 transcripts were expressed at some level in the urine of both BCa patients and healthy volunteers, with some showing similar expression levels in patients and controls while others appeared differentially expressed, but the difference was not always statistically confirmed (Figure 1A). One circRNA-circSTAG2(16–25), also known as hsa_circ_0139697, a 1117 nt transcript that has the end of exon 25 back spliced to the beginning of exon 16 was selected for further study (Figure 1B). This circRNA was selected because its expression was significantly increased in urine derived from BCa patients compared to the urine obtained from control subjects.

**Figure 2:**
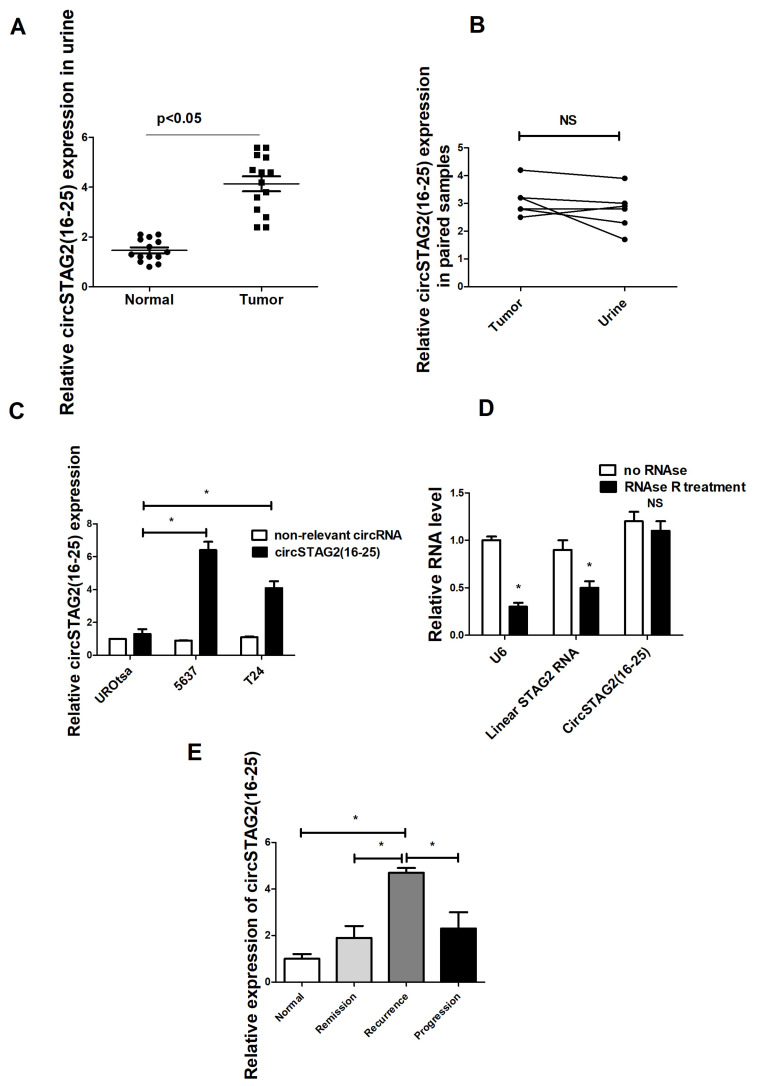
CircSTAG2(16–25) in BCa: (**A**) Relative circSTAG2(16–25) expression in the urine of BCa patients and control group (14 samples in each group). (**B**) circSTAG2(16–25) expression in pairs of urine-tissue samples, (**C**) circSTAG2(16–25) expression in BCa cell lines, (**D**) RNase R assay to prove the circular structure of circSTAG2(16–25), (**E**) circSTAG2(16–25) is expressed at higher levels in urine samples related to recurrent tumor. For (**C**,**D**), NS—non-significant difference, data are shown as the means ± SD of three independent experiments, * *p* < 0.05.

To evaluate the potential significance of circSTAG2(16–25) in BCa, 14 BCa samples and 14 controls (7 males, 7 females, age-matched) were analyzed (Figure 2A). Additional control and BCa samples were examined. For some tested urine samples, RT-qPCR results were unreliable possibly because of low quality and/or level of isolated RNA. CircSTAG2(16–25) levels were nonetheless generally higher in the urine of BCa patients. To explore whether the source of circSTAG2(16–25) isolated from urine could indeed be the bladder tumor of the patient, we compared circSTAG2(16–25) levels in seven paired samples of urine and tumor tissue. In five tested pairs, circSTAG2(16–25) was detected in both paired samples (Figure 2B). In the other two samples, the level of circRNA in urine was under the limit of detection.

We further measured circSTAG2(16–25) levels using RT-qPCR in several urothelial cell lines and found that its expression levels were higher in 5637 and T24 cells, which are aggressive BCa cell lines, compared to non-transformed urothelial cells (UROtsa) (Figure 2C). Another circRNA derived from STAG2 (hsa_circ_0091460) was used as a control to compare expressions. As expected, circSTAG2(16–25) was resistant to digestion by the exonuclease RNase R, unlike linear RNAs like U6 snRNA and STAG2 mRNA that were, at least partly, digested (Figure 2D).

### 3.2. CircSTAG2(16–25) Is Expressed at Higher Levels in Urine in Recurrent Samples

To examine circSTAG2(16–25) expression relative to clinical status, we compared its expression in urine from patients with different clinical histories. The clinical course of each bladder cancer patient was reviewed. Patient clinical courses were separated into remission, recurrence, or progression categories. Remission was defined as the absence of bladder cancer while patients remained on surveillance status post-surgery for bladder cancer. Recurrence was defined as the identification of bladder cancer of equal or lower grade and stage on surveillance after initial bladder cancer treatment. Progression was defined as the upstaging of bladder cancer to a higher grade or stage on subsequent surveillance. Bladder cancer surgeries were performed in the standard transurethral technique. Ten samples (age 55–70) were selected from each group. CircSTAG2(16–25) was detected at ~5× fold higher levels in urine derived from patients with recurrent tumors as compared with healthy samples. It was ~2.0× fold higher in urine derived from patients with remission and ~1.7× fold higher in urine samples derived from patients with potential BCa progression (Figure 2E).

### 3.3. Knockdown of circSTAG2(16–25) Inhibited BCa Cell Proliferation, While circSTAG2(16–25) Overexpression Promoted It

**Figure 3:**
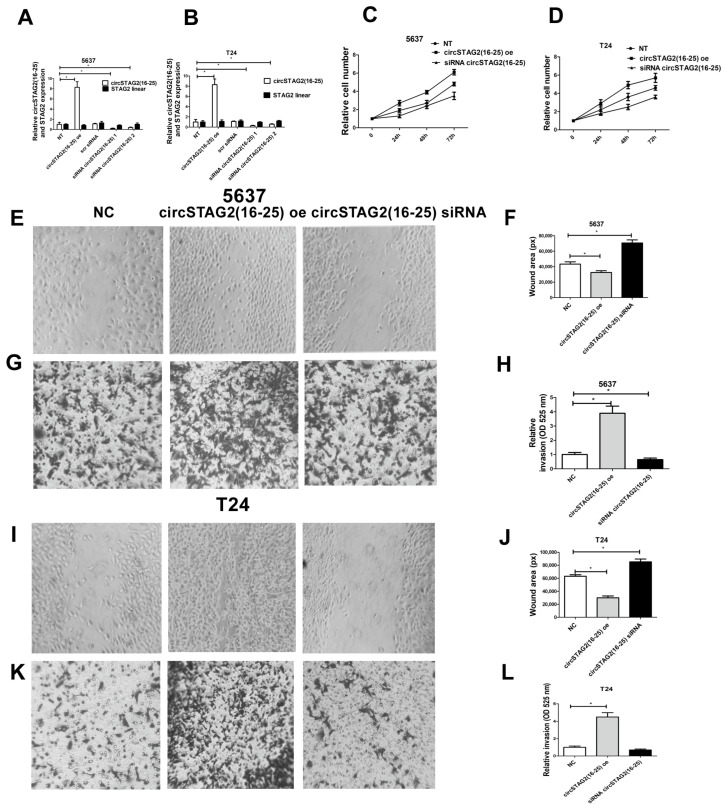
CircSTAG2(16–25) regulates BCa cell proliferation, motility, and invasion. (**A**,**B**) efficiency of manipulating circSTAG2(16–25) expression in 5637 and T24 cells. (**C**,**D**) Cell proliferation depends on circSTAG2(16–25), (**E**,**F**) circSTAG2(16–25) regulates cell motility in 5637 cells; (**G**,**H**) Cell invasion depends on circSTAG(16–25) in 5637 cells. (**I**,**J**) circSTAG2(16–25) regulates cell motility in T24 cells; (**K**,**L**) Cell invasion depends on circSTAG(16–25) in T24 cells. Magnification is 100×. For graphs, data are shown as the means ± SD of three independent experiments, * *p* < 0.05.

To evaluate the biological roles of circSTAG2(16–25) in BCa, we designed two siRNAs targeting the circSTAG2(16–25) back-splicing junction (see Section 2) and transfected them into 5637 and T24 cells. Compared to a non-relevant scrambled siRNA, both siRNAs knocked down circSTAG2(16–25) in 5637 and T24 cells by several folds (Figure 3A,B). We also generated circSTAG2(16–25) overexpression plasmids by inserting STAG2 exons 16 to 25 in between the previously described ZKSCAN1 introns that drive high levels of back splicing [27]. RT-qPCR confirmed transfection of this plasmid increased circSTAG2(16–25) levels by ~8-fold in 5637 and T24 cells (Figure 3A,B). Transfection of a control plasmid (empty vector) did not change circSTAG2(16–25) expression. Importantly, linear STAG2 mRNA expression did not change substantially after transfection of siRNA or overexpression plasmid (Figure 3A,B). We then used the siRNAs and overexpression plasmids to explore the roles of circSTAG2(16–25) on BCa cell behavior: proliferation, motility, and matrix invasion. In both 5637 and T24 cell lines, silencing of circSTAG2(16–25) with siRNA 1 inhibited cell proliferation. In contrast, overexpression of circSTAG2(16–25) caused elevated proliferation (Figure 3C,D) in 5637 and T24 cells.

Cell motility was increased for circSTAG2(16–25) overexpressing 5637 and T24 cells whereas silencing of circSTAG2(16–25) with siRNA 1 partly inhibited motility in both cell lines (Figure 3E,F,I,J).

Matrix invasion was also increased in circSTAG(16–25) overexpressing 5637 and T24 cells. Transfection with siRNA 1 circSTAG2(16–25) induced diminishing invasion of 5637 cells. T24 cells invaded less efficiently when transfected with siRNA 1 circSTAG2(16–25), but this difference was not statistically significant in these cell lines (Figure 3G,H,K,L).

### 3.4. CircSTAG2 May Act as a Sponge for miR-145-5p in BCa Cells

**Figure 4:**
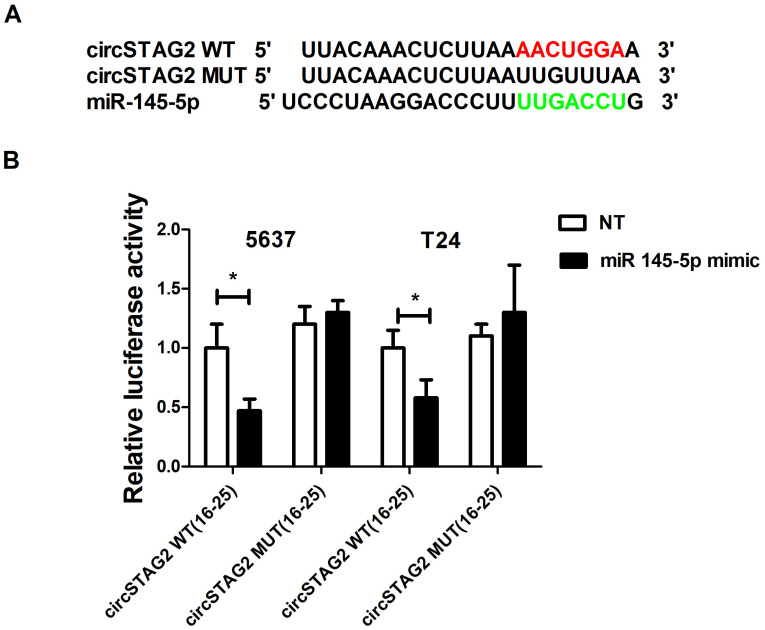
circSTAG2 -miR-145-p interactions. (**A**) Potential miR-145-5p binding motif, (**B**) Dual-luciferase reporter gene assays were carried out to determine the potential for an interaction between circSTAG2(16–25) and miR-145-5p in 5637 andT24. Red and green sequences are binding motifs from circSTAG2(16-25) and miR-145-5p accordingly. Data are shown as the means ± SD of three independent experiments, * *p* < 0.05.

Some circRNAs have been suggested to interact with and sponge miRNAs, so we used the Circular RNA interactome database to identify potential miRNA binding motifs within circSTAG2(16–25). Among potential candidates, miR-145-5p was chosen for further research as circSTAG2(16–25) possesses two potential miR-145-5p binding sites. In addition, miR-145-5p has previously been linked to BCa biology. MiR-145-5p behaves as a tumor suppressor in BCa and has been shown to reduce cell motility and invasion in in vitro assays [20,33,34]. Figure 4A highlights one of the predicted binding sites (with the highest binding score) of miR-145-5p with circSTAG2(16–25). The binding of this region of circSTAG2(16–25) with miR-145-5p was verified using luciferase reporter assays. Luciferase activity from a reporter containing the wild-type circSTAG2(16–25) region was inhibited when a ~50% excess of miR-145-5p was present in both cell lines. In contrast, there was no change in luciferase activity when the miR-145-5p binding site was mutated (Figure 4B).

### 3.5. miR-145-5p Expression Is Correlated with BCa Progression and It Suppresses Proliferation, Migration, and Invasion of BCa Cells

**Figure 5:**
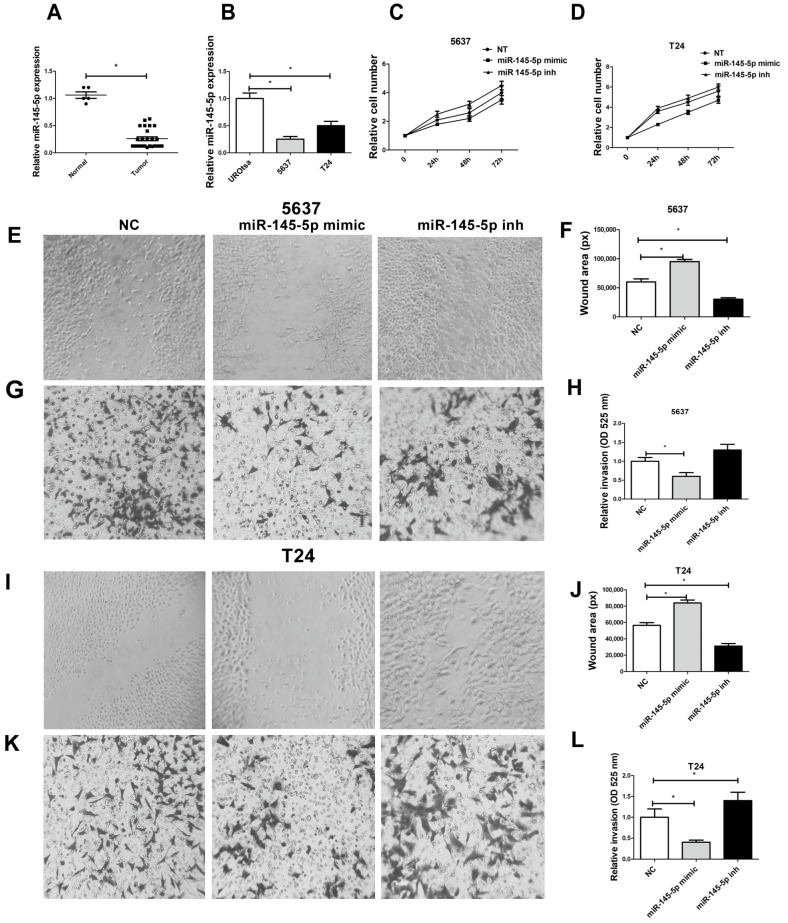
miR-145-5p regulates BCa cells proliferation, motility, and invasion: (**A**) miR-145-5p expression in urine samples. (**B**) miR-145-5p expression in urothelial cell lines. (**C**,**D**) miR-145-5p regulates BCa cell proliferation in 5637 and T24 cell lines. (**E**,**F**) miR-145-5p regulates 5637 cell motility. (**G**,**H**) miR-145-5p regulates 5637 cell invasion. (**I**,**J**) miR-145-5p regulates T24 cell motility, (**K**,**L**) miR-145-5p regulates 5637 cell invasion. Magnification is 100×. Data are shown as the means ± SD of 3 independent experiments, * *p* < 0.05.

Using RT-qPCR, we found suppressed expression of miR-145-5p in the urine of BCa patients and in more aggressive BCa cell lines (Figure 5A,B). The 5637 and T24 cell proliferation activity was modulated by miR-145-5p expression (Figure 5C,D). Mobility of 5637 (Figure 5E,F) and T24 cells (Figure 5I,J) was dependent on the levels of miR-145-5p. For 5637 cells, a trend of increasing invasion after transfection with miR-145-5p inhibitor was observed (Figure 5G,H). Matrix invasion was inhibited with an excess of miR-145-5p, whereas inactivation of miR-145-5p induced elevated invasion activity in T24 cells (Figure 5K,L).

### 3.6. miR-145-5p Suppressed BCa Cell Proliferation, Migration, and Invasion by Targeting TAGLN2

**Figure 6:**
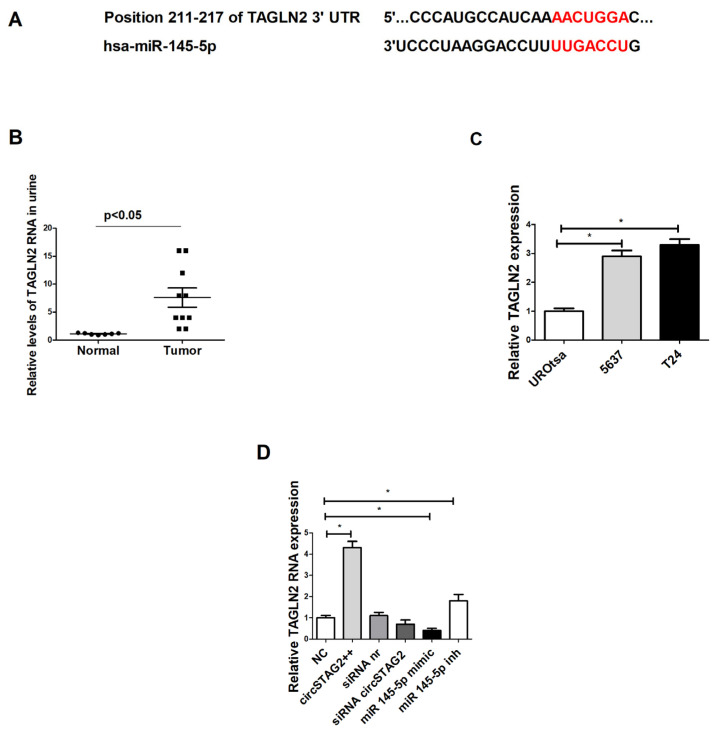
TAGLN2 is regulated by circSTAG2(16–25) via miR-145-5p: (**A**) TAGLN2 is a possible downstream target of miR-145-5p, (**B**) Relative expression of TAGLN2 mRNA in urine samples, (**C**) Relative expression of TAGLN2 mRNA in urothelial cell lines, (**D**) TAGLN2 RNA expression is regulated by circSTAG2(16–25) and miR-145-5p levels. Red-binding motifs of TAGLN2 and miR-145-5p. For (**C**,**D**) data are shown as the means ± SD of three independent experiments, * *p* < 0.05.

miR-145-5p was identified as a tumor suppressor gene in BCa [20,29]. To choose potential targets that can be regulated by miR-145-5p, we used the miRBD database and software (https://mirdb.org/, accessed on 2 June 2023). For the current study, we selected the TAGLN2 gene because of its high binding score to miR-145-5p (Figure 6A) and its known ability to be regulated by miR-145p in BCa [20]. This regulation plays an important role in BCa development and progression [20,35,36,37]. Like circSTAG2(16–25), TAGLN2 mRNA contains a binding site for the seed of miR-145-5p (Figure 6A). Therefore, we aimed to study whether there is a potential relationship between circSTAG2(16–25), miR-145-5p, and TAGLN2 in relation to BCa biology.

We found that TAGLN2 was overexpressed in tumor urine samples as compared with control (Figure 6B). TAGLN2 RNA levels were also elevated in BCa cell lines with higher malignant potency (Figure 6C). CircSTAG(16–25) overexpression in 5637 cells induced elevation of TAGLN2 expression ~4.5× fold. Suppression of circSTAG2(16–25) expression by siRNA modulated TAGLN2 down-regulation (not statistically proven) (Figure 6D). miR-145-5p overexpression also induced TAGLN2 downregulation, whereas inhibition of miR-145-5p activity caused an increase in TAGLN2 mRNA expression (Figure 6D).

Therefore circSTAG2(16–25) may regulate BCa cell behavior by modulating the activity of miR-145-5p. Reduced levels of free miR-145p inside cells may induce activation of TAGLN2 and as a result- an increase in the malignant activity of cells.

## 4. Discussion

**Figure 7:**
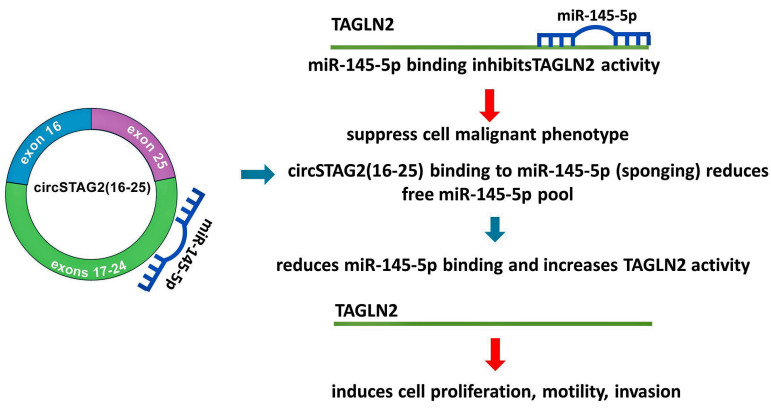
The schematic diagram for a possible mechanism connecting circSTAG2(16–25), miR-145-5p, and TAGLN2 activity in BCa.

For the current study, circSTAG2(16–25) was selected from circRNAs previously determined and validated as circRNAs derived from the STAG2 gene [38]. There are no data regarding the expression of these molecules in the urothelium. Therefore, we detected and validated several circSTAG2 isoforms in BCa cells, tumors, and in the patient’s urine. Based on the bioinformatic analysis and the data from preliminary experiments, we selected one of the tested circSTAG2 isoforms since its expression was clearly altered in samples derived from different tumors and cells. We next found that the levels of circSTAG2(16–25) were higher in urine samples derived from patients with recurrent disease. The ability to detect this molecule in a patient’s urine samples is critical for consideration as a potential biomarker. We were able to detect it in a small amount of urine (1 mL) despite its known low abundance. This is likely because of the high stability of circRNAs in biologically active environments. To prove the circular structure of this RNA, we performed sequencing across the back splicing junction and RNase R assay. Manipulation of circSTAG2(16–25) expression in BCa cells showed direct dependency of cells behavior on circSTAG2(16–25) levels. It was previously shown that STAG2 downregulation in T24 cells induced elevation of invasion but not motility [39]. CircSTAG2(16–25) regulates at least partly both mechanisms, using different signaling pathways. As a target for circSTAG2(16–25) in BCa cells, we tested miR-145-5p. This choice was dictated by the high binding predicted score for circSTAG2(16–25) and miR-145-5p. In addition, miR-145-5p was able to downregulate the potential BC-related oncogene TAGLN2 in BCa cells [20]. In this study, we showed that the expression of TAGLN2 can be affected by circSTAG2(16–25) expression (Figure 7). Therefore, a network consisting of circSTAG2(16–25), miR-145-5p, and TAGLN2 represents a possible mechanism of BCa cell proliferation, motility, and invasion regulation (Figure 7). Currently, there are no commercially approved biomarker tests based on the use of circRNA. It is not exactly known yet what is the amplitude of the circRNA activity in gene regulation. However, its stability and relatively easy detection in active environments such as biological fluids make circRNAs potentially good candidates for a non-invasive biomarker. Although it is preliminary to consider circSTAG2(16–25) as a potential biomarker for non-invasive analysis of clinical status in BCa, further studies using follow-up observations may confirm that circSTAG2(16–25) can be considered as a potential marker for the prediction of recurrence in BCa.

Animal study is an important part of cancer biology research. There are several types of BCa animal models that can be developed in rodents. These models include (i) orthotopic (the tumor grows in the urothelium) and (ii) heterotopic (the tumor grows outside the bladder). In addition, based on the type of tumor-forming cells, models can be divided into three types: (a) xenogeneic (implantation of human bladder cancer cells or tissue into immunodeficient mice), (b) syngeneic (implantation of murine cells), and (c) transgenic (genetic modification of experimental animal) [40]. In this study, we were interested in a human circular RNA isolated from urine of BCa patients that originated from BCa tumors, so the orthotopic xenogeneic model is the only logical option to support the concept of this study. Heterotopic models do not provide direct contact of the tumor with urine, and the circular RNA of interest may not be produced in an orthotopic mouse model. It is possible to establish orthotopic implantation of human BC cultured cells in the bladder wall [41]. The tumor grows relatively fast, and cells may be modified before implantation. Future results related to human circSTAG2(16–25) biology might thus be obtained by using cultured BC cells in this model, but it is generally much more complicated to implant and grow human BC tissue in rodent urothelium.

## 5. Conclusions

This work revealed tumorigenic effects and potential mechanisms of action of the circRNA circSTAG2(16–25). Elevated circSTAG2(16–25) levels in BCa indicated that it could be considered a potential diagnostic or prognostic biomarker.

## Figures and Tables

**Table 1:** Primers for RT-qPCR.

Template		Primer 5′–3′
hsa_circ_0091459 circ 1	Forward Reverse	GTCCAAACCGAATGAATGG TGGTAAAATGGTGGCTTGC
hsa_circ_0091460 circ 2	Forward Reverse	CAGTCGGTGGTAGATGATTGGA GTGGCTTGCCTTCTTTCTTCT
hsa_circ_0139698 circ 3	Forward Reverse	TCATTGCAGTCTGAGTTGCTC CACGTTGGTCAGGTAATGTTG
hsa_circ_0091464 circ 4	Forward Reverse	AGAACTTGCTCGACGTTTTGC TGGCCGGTGAGCTGAATAAA
hsa_circ_0139699 circ 5	Forward Reverse	GCCATTGCCATGCTACACAA TGAAGGCCTGTTCCTTAACAGTAG
hsa_circ_0139701 circ 6	Forward Reverse	GGGGGTCAACAGTACGGAGT CACGCCCTCCTGACATAATC
hsa_circ_0139700 circ 7	Forward Reverse	CTAATCCGCAAGGGGAGAG TCTTTAAACGCAGCAAGTCC CAGCTCAGTAGCACAGTCCC
circSTAG2(16–25)	Forward Reverse	TCATTGCAGTCTGAGTTGCT CAGCTCAGTAGCACAGTCCC
hsa_circ_0139702 circ 9	Forward Reverse	CTAATCCGCAAGGGGAGAG AAGCAAAACGTCGAGCAAGT
miR-145-5p	Forward Reverse	CACGCGGTCCAGTTTTCC CCAGTGCAGGGTCCGAGGTA
U6	Forward Reverse	GCTTCGGCAGCACATATACTAAAAT CGCTTCACGAATTTGCGTGTCAT
TAGLN2	Forward Reverse	AGAACCCTGAAGTCCTCCCT ATATGCAGGTCCCCTGTTGG
β-ACT	Forward Reverse	CACCATTGGCAATGAGCGGTTC AGGTCTTTGCGGATGTCCACGT

## Data Availability

The datasets generated and/or analyzed during this current study are not publicly available due to the need for further research but are available from the corresponding author upon reasonable request.
